# Supplementary figures and images for: Genome-wide identification and characterization of WRKY gene family in Salix suchowensis
Source: PeerJ. 2016 Sep 7;4:e2437. doi: 10.7717/peerj.2437 (PMC5018666; doi:10.7717/peerj.2437)

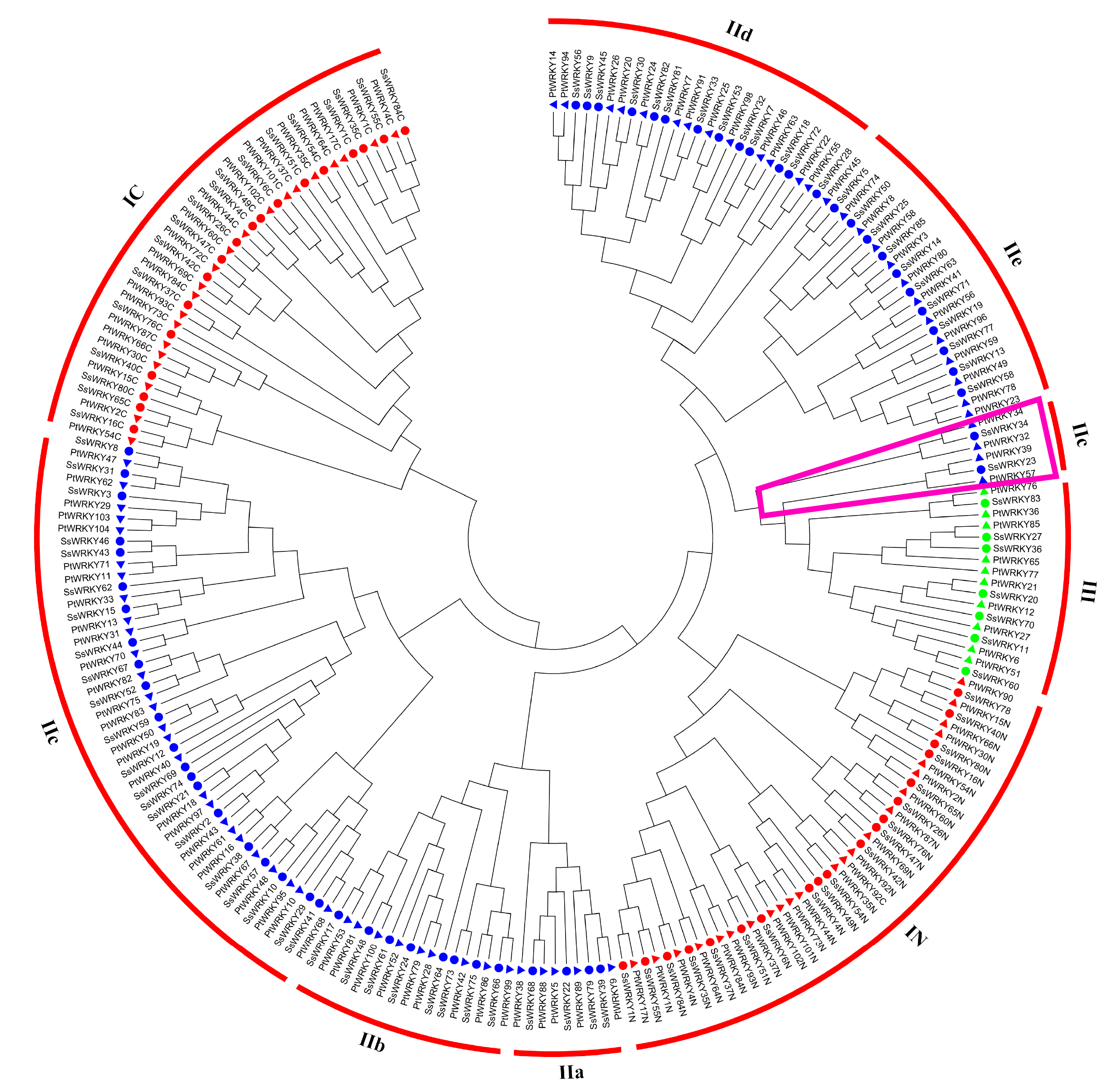

Supplement: Supplemental Information 1 — The phylogenetic tree was constructed using the neighbor-joining method in MEGA 6.0. The WRKY genes with the suffix ‘N’ and ‘C’ indicate the N-terminal and the C-terminal WRKY domains of group I, respectively. The different colors indicate different groups (I, II and III) or subgroups (IIa, b, c, d and e) of WRKY domains. Circles indicate WRKY genes from willow, and triangles represent genes from poplar. The purple trapezoid region indicate a new subgroup belonging to IIc. [file peerj-04-2437-s001.png]

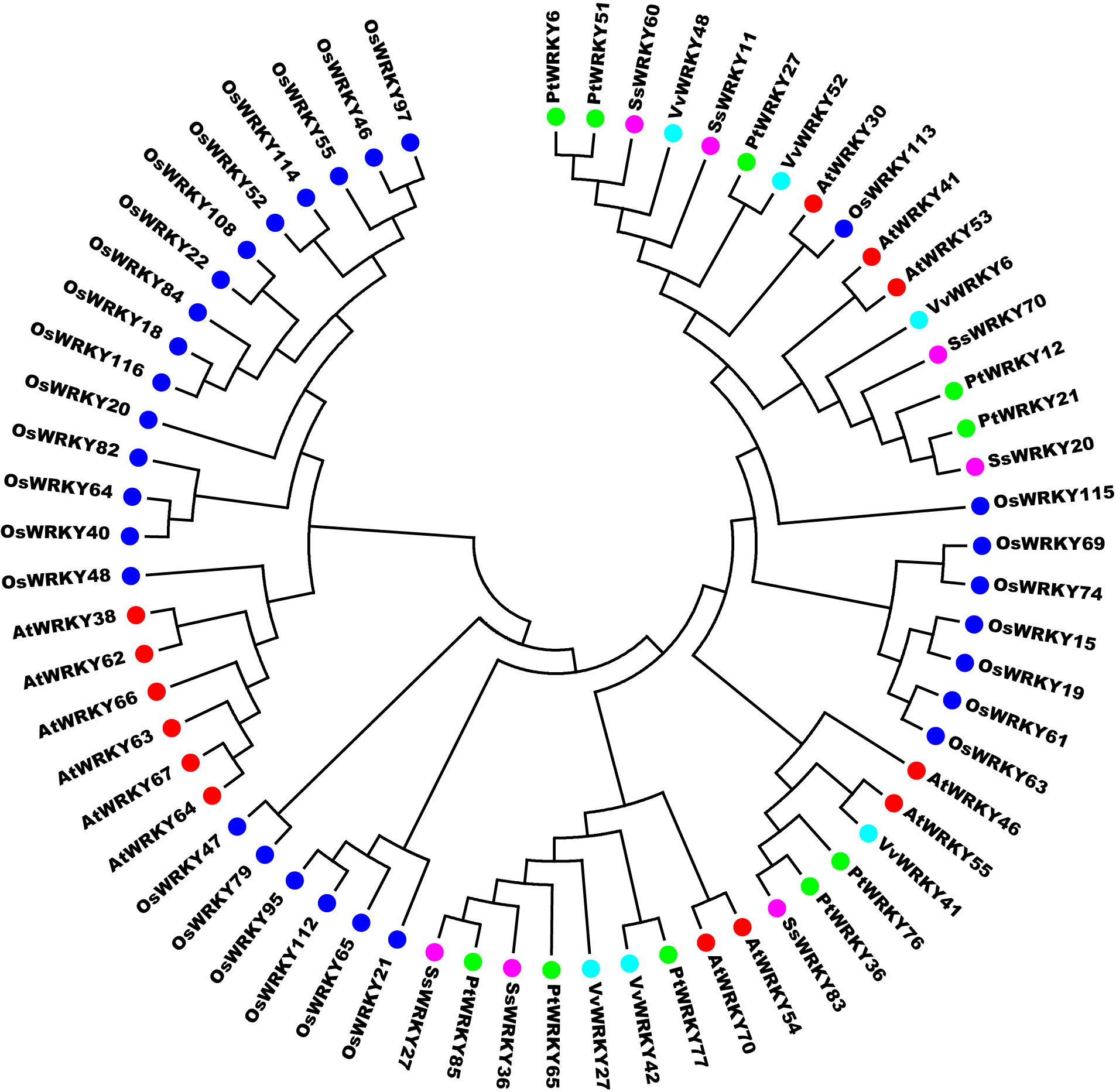

Supplement: Supplemental Information 2 — The phylogenetic tree was constructed using the neighbor-joining method in MEGA 6.0. Dicotyledonous (Arabidopsis, grape, poplar and willow) and monocotyledonous (rice) WRKY III genes are marked with colored dots. [file peerj-04-2437-s002.png]

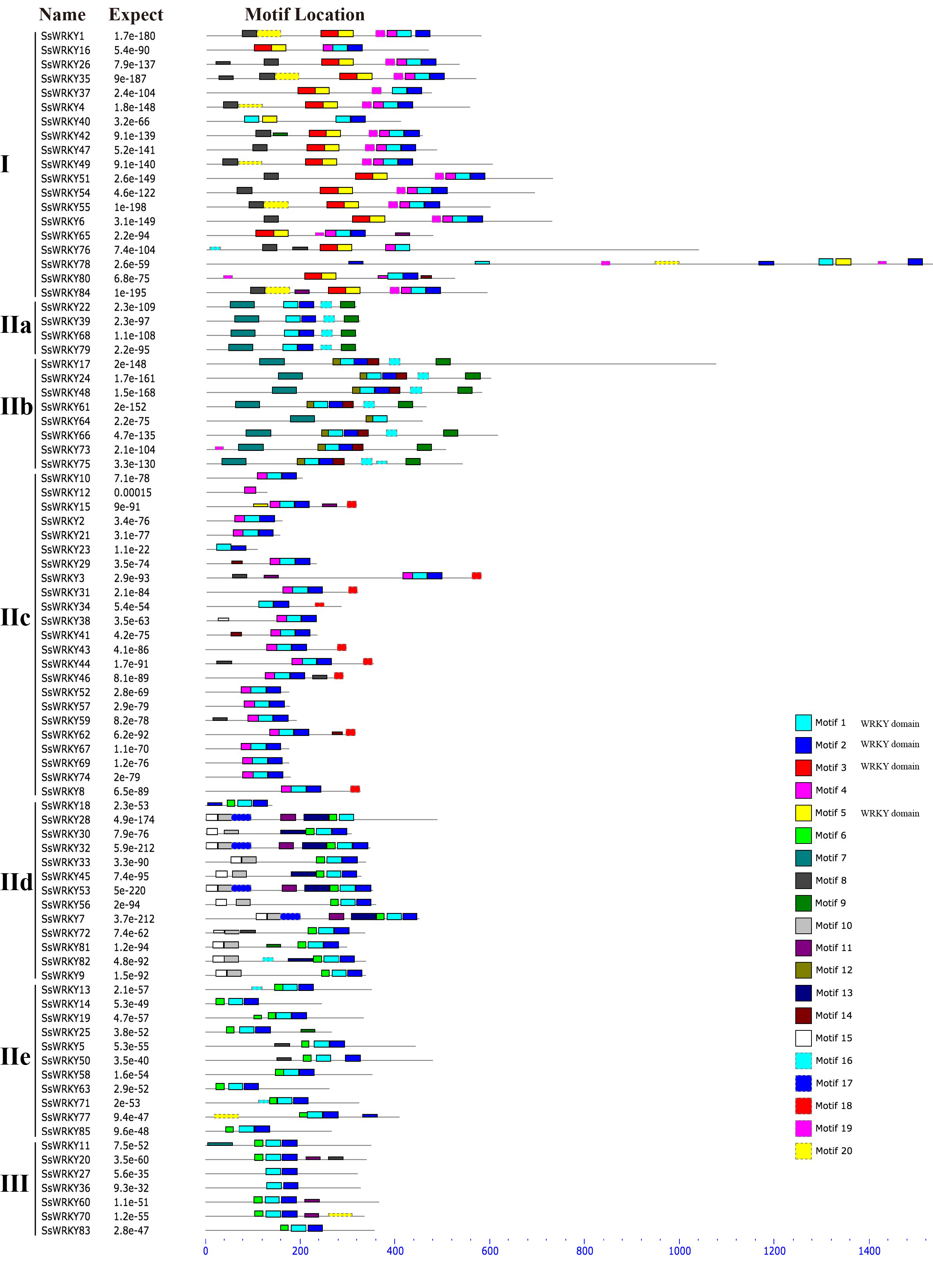

Supplement: Supplemental Information 3 — The names of all members are displayed on the left side of the figure. Different motifs are displayed in different colored boxes as indicated on the right side. The conserved motifs 1, 2, 3, and 5, broadly distributed across SsWRKY genes, were definitely characterized as the WRKY conserved domains. [file peerj-04-2437-s003.png]
