# Supplementary material for: Genome-wide identification and characterization of WRKY gene family in Salix suchowensis
Source: PeerJ. 2016 Sep 7;4:e2437. doi: 10.7717/peerj.2437 (PMC5018666; doi:10.7717/peerj.2437)
Supplement: Supplemental Information 4 [file peerj-04-2437-s004.docx]

**Table S1. The Ka/Ks ratios of 21 WRKY III gene pairs in *Salix suchowensis*.**

| No. | WRKY III Gene pairs | Ks | Ka | Ka/Ks |
| --- | --- | --- | --- | --- |
| 1 | SsWRKY11/27 | 2.4870 | 0.9915 | 0.3987 |
| 2 | SsWRKY11/60 | 0.4007 | 0.1665 | 0.4155 |
| 3 | SsWRKY20/11 | 4.3128 | 0.4394 | 0.1019 |
| 4 | SsWRKY20/27 | 54.0428 | 0.9482 | 0.0175 |
| 5 | SsWRKY20/36 | 51.6711 | 0.9860 | 0.0191 |
| 6 | SsWRKY20/60 | 4.3851 | 0.3692 | 0.0842 |
| 7 | SsWRKY20/70 | 0.3845 | 0.1249 | 0.3248 |
| 8 | SsWRKY20/83 | 5.7710 | 1.0414 | 0.1805 |
| 9 | SsWRKY27/60 | 4.5878 | 1.0489 | 0.2286 |
| 10 | SsWRKY36/11 | 48.0562 | 1.1912 | 0.0248 |
| 11 | SsWRKY36/27 | 0.7402 | 0.2150 | 0.2905 |
| 12 | SsWRKY36/60 | 4.0536 | 1.1207 | 0.2765 |
| 13 | SsWRKY36/70 | 2.9280 | 1.1635 | 0.3974 |
| 14 | SsWRKY36/83 | 51.7188 | 1.0382 | 0.0201 |
| 15 | SsWRKY70/11 | 5.1184 | 0.4757 | 0.0929 |
| 16 | SsWRKY70/27 | 52.9026 | 1.0387 | 0.0196 |
| 17 | SsWRKY70/60 | 2.5862 | 0.4203 | 0.1625 |
| 18 | SsWRKY70/83 | 52.8751 | 0.9805 | 0.0185 |
| 19 | SsWRKY83/11 | 47.6615 | 0.9353 | 0.0196 |
| 20 | SsWRKY83/27 | 53.7379 | 1.0215 | 0.0190 |
| 21 | SsWRKY83/60 | 11.7730 | 0.9260 | 0.0787 |
