# Supplementary material for: Genome-wide identification and characterization of WRKY gene family in Salix suchowensis
Source: PeerJ. 2016 Sep 7;4:e2437. doi: 10.7717/peerj.2437 (PMC5018666; doi:10.7717/peerj.2437)
Supplement: Supplemental Information 5 [file peerj-04-2437-s005.docx]

**Table 2. The details of twenty conserved motif sequences identified in SsWRKY genes.**

| Motif | Width | Best possible match |
| --- | --- | --- |
| 1^*^ | 29 | ILDDGYRWRKYGQKVIKGNPYPRSYYRCT |
| 2^*^ | 29 | CPVRKHVERCWEDPTMVITTYEGEHNHPW |
| 3^*^ | 37 | PSDDGYNWRKYGQKQVKGSEYPRSYYKCTHPNCPVKK |
| 4 | 21 | KKGHKKIREPRFAFQTRSEVD |
| 5^*^ | 29 | KVECSHDGHITEIIYKGTHNHPKPQPNCR |
| 6 | 15 | KRRKNRVKWVVRVPA |
| 7 | 50 | KEELAVLQEELNRMKEENKRLKEMLDQICENYNALQMHFMDLMQQNNEKH |
| 8 | 29 | PVIRSPYFTIPPGLSPTELLDSPVFFSNS |
| 9 | 29 | LVEQMTAAITADPNFTAALAAAISGIMGQ |
| 10 | 28 | QVQYRNCMVITDETVFKFKKVISLLNRT |
| 11 | 29 | LQQQQQQQMKYQADMMYRKSNSGINLNFD |
| 12 | 15 | MRKARVSVRARCEAP |
| 13 | 50 | MDGTVANLDGDAFHLMGMPHSSDHISQQHKRKCSGRGEDGNVKCGSSGKC |
| 14 | 21 | PPAAMAMASTTSAAASMLLSG |
| 15 | 21 | VEEAARAGIESCEHVIRLLCQ |
| 16 | 21 | MATISASAPFPTITLDLTQNP |
| 17 | 40 | LGHGRVRKLKKLPSHLPQNIFLDNPHCKTIHAPKPPQMVP |
| 18 | 17 | LLPDYGLLQDIVPSHMH |
| 19 | 17 | GGEDDEDEPEPKRWKIE |
| 20 | 49 | PSPTTGTFPGQAFNWKSNSGDNQQGVKGEDKDFSDFSFQTPARPPATSS |

^*^, WRKY conserved domains.
