# Supplementary material for: Genome-wide identification and characterization of WRKY gene family in Salix suchowensis
Source: PeerJ. 2016 Sep 7;4:e2437. doi: 10.7717/peerj.2437 (PMC5018666; doi:10.7717/peerj.2437)
Supplement: Supplemental Information 6 [file peerj-04-2437-s006.docx]

**Table S3. The details of 33 homologus SsWRKY gene pairs in *Salix suchowensis*.**

| No. | Gene name | Homologous gene | Group | Identity (%) |
| --- | --- | --- | --- | --- |
| 1 | SsWRKY1 | SsWRKY35 | I | 72.38 |
| 2 | SsWRKY2 | SsWRKY74 | IIc | 74.85 |
| 3 | SsWRKY4 | SsWRKY49 | I | 81.56 |
| 4 | SsWRKY5 | SsWRKY50 | IIe | 68.79 |
| 5 | SsWRKY6 | SsWRKY51 | I | 80.92 |
| 6 | SsWRKY7 | SsWRKY32 | IId | 91.17 |
| 7 | SsWRKY8 | SsWRKY31 | IIc | 83.08 |
| 8 | SsWRKY9 | SsWRKY56 | IId | 79.44 |
| 9 | SsWRKY10 | SsWRKY57 | IIc | 84.44 |
| 10 | SsWRKY11 | SsWRKY60 | III | 67.84 |
| 11 | SsWRKY12 | SsWRKY59 | IIc | 74.22 |
| 12 | SsWRKY13 | SsWRKY58 | IIe | 83.19 |
| 13 | SsWRKY14 | SsWRKY63 | IIe | 78.93 |
| 14 | SsWRKY15 | SsWRKY62 | IIc | 79.26 |
| 15 | SsWRKY16 | SsWRKY65 | I | 73.49 |
| 16 | SsWRKY17 | SsWRKY64 | IIb | 67.57 |
| 17 | SsWRKY19 | SsWRKY71 | IIe | 73.93 |
| 18 | SsWRKY20 | SsWRKY70 | III | 77.55 |
| 19 | SsWRKY21 | SsWRKY69 | IIc | 77.48 |
| 20 | SsWRKY22 | SsWRKY68 | IIa | 81.06 |
| 21 | SsWRKY24 | SsWRKY48 | IIb | 79.45 |
| 22 | SsWRKY25 | SsWRKY85 | IIe | 81.48 |
| 23 | SsWRKY26 | SsWRKY76 | I | 77.46 |
| 24 | SsWRKY27 | SsWRKY36 | III | 66.17 |
| 25 | SsWRKY28 | SsWRKY53 | IId | 85.80 |
| 26 | SsWRKY29 | SsWRKY41 | IIc | 83.12 |
| 27 | SsWRKY30 | SsWRKY45 | IId | 83.54 |
| 28 | SsWRKY33 | SsWRKY81 | IId | 80.46 |
| 29 | SsWRKY39 | SsWRKY79 | IIa | 66.37 |
| 30 | SsWRKY42 | SsWRKY47 | I | 73.74 |
| 31 | SsWRKY43 | SsWRKY46 | IIc | 80.00 |
| 32 | SsWRKY52 | SsWRKY67 | IIc | 74.16 |
| 33 | SsWRKY55 | SsWRKY84 | I | 81.05 |
