# Supplementary material for: Genome-wide identification and characterization of WRKY gene family in Salix suchowensis
Source: PeerJ. 2016 Sep 7;4:e2437. doi: 10.7717/peerj.2437 (PMC5018666; doi:10.7717/peerj.2437)
Supplement: Supplemental Information 7 [file peerj-04-2437-s007.docx]

**Table S1. The details of total transcript abundance of SsWRKY genes by RPKM annotation.**

| **Name** | **Root** | **Stem** | **Bark** | **Bud** | **Leaf** |
| --- | --- | --- | --- | --- | --- |
| SsWRKY1 | 2.023720073 | 0.071695055 | 1.180604052 | 2.35163719 | 0.084382916 |
| SsWRKY2 | 1.603504485 | 0.385305942 | 71.60874106 | 10.08050613 | 0 |
| SsWRKY3 | 5.37117394 | 3.131296866 | 7.300974757 | 3.238454218 | 1.347818751 |
| SsWRKY4 | 26.17155936 | 30.32023512 | 28.39766839 | 51.22326306 | 10.46789379 |
| SsWRKY5 | 28.62649425 | 3.250550933 | 1.573264179 | 0.632350871 | 0 |
| SsWRKY6 | 25.66955935 | 22.2540866 | 15.66114503 | 17.96716263 | 12.31990577 |
| SsWRKY7 | 11.3389358 | 8.738269159 | 7.069172254 | 6.603712458 | 4.468453341 |
| SsWRKY8 | 1.657964727 | 0.349976471 | 4.858968307 | 1.900813126 | 0.024964348 |
| SsWRKY9 | 11.94887661 | 22.18181755 | 45.88708649 | 12.08178308 | 10.33905818 |
| SsWRKY10 | 0.221735933 | 6.050712948 | 0.744088891 | 0.538336458 | 0.320517874 |
| SsWRKY11 | 1.549631804 | 1.73962349 | 1.664053337 | 0.923141669 | 1.073325124 |
| SsWRKY12 | 6.862087502 | 0.080519062 | 4.787353448 | 0.754591274 | 0 |
| SsWRKY13 | 12.73214951 | 0.741322811 | 0.864239222 | 4.533158982 | 1.000483377 |
| SsWRKY14 | 12.8990185 | 8.462618379 | 31.76239442 | 33.57246561 | 8.643804854 |
| SsWRKY15 | 30.55841634 | 7.523499881 | 37.37186454 | 45.23568354 | 3.824304083 |
| SsWRKY16 | 13.83857239 | 11.47439195 | 11.98376396 | 9.799316915 | 8.178542096 |
| SsWRKY17 | 7.283651398 | 6.573614941 | 7.725595747 | 7.740314143 | 5.290803648 |
| SsWRKY18 | 10.80768848 | 3.733156523 | 20.43798843 | 16.84968015 | 0.861531872 |
| SsWRKY19 | 4.633522081 | 0 | 0.32686762 | 0.985347981 | 0.09777703 |
| SsWRKY20 | 1.938169225 | 0.549313719 | 4.963051208 | 4.307825185 | 0.191563161 |
| SsWRKY21 | 23.91467647 | 1.921245979 | 52.17193386 | 64.91932603 | 16.21861013 |
| SsWRKY22 | 34.92384117 | 11.8044457 | 20.62354785 | 28.24868852 | 7.727126775 |
| SsWRKY23 | 0.103308787 | 0.523373905 | 0.221873778 | 1.22621082 | 5.002628403 |
| SsWRKY24 | 23.38535268 | 1.418732568 | 21.67303407 | 19.96392823 | 2.239982867 |
| SsWRKY25 | 51.62548243 | 0.644452942 | 6.556866882 | 6.108177034 | 11.24727716 |
| SsWRKY26 | 4.488555567 | 5.009991839 | 8.471825449 | 8.136752098 | 5.434803536 |
| SsWRKY27 | 2.472714947 | 1.356895309 | 12.03357089 | 6.149977108 | 20.53317628 |
| SsWRKY28 | 4.871924566 | 12.3541824 | 3.529193908 | 10.7511967 | 11.74280224 |
| SsWRKY29 | 3.129906045 | 16.92094446 | 2.8697869 | 2.935631837 | 1.218069779 |
| SsWRKY30 | 2.978016961 | 1.396785662 | 9.377912592 | 2.42482204 | 0.13204615 |
| SsWRKY31 | 1.354528522 | 0.307867003 | 6.328211092 | 1.423619373 | 0 |
| SsWRKY32 | 25.09813187 | 22.71442747 | 39.67737082 | 28.34298725 | 3.003710359 |
| SsWRKY33 | 43.90167671 | 13.66929492 | 6.209202944 | 7.140874777 | 7.077906648 |
| SsWRKY34 | 2.012369079 | 0.817771726 | 0.508460742 | 0.659939851 | 0.228146403 |
| SsWRKY35 | 2.647625718 | 2.173874159 | 52.63500414 | 5.884955944 | 0.530350801 |
| SsWRKY36 | 4.889949249 | 0.792990765 | 11.96269455 | 0.520210651 | 0.273775684 |
| SsWRKY37 | 5.848053775 | 19.62378983 | 14.89078766 | 13.87486983 | 6.532893665 |
| SsWRKY38 | 0 | 0.089465625 | 0.834397115 | 0.052402172 | 0 |
| SsWRKY39 | 0.502370473 | 0.494652166 | 5.394644458 | 3.588909718 | 1.302103862 |
| SsWRKY40 | 4.035031606 | 3.792564527 | 5.114083405 | 5.716393438 | 3.848730627 |
| SsWRKY41 | 4.123633437 | 21.22203892 | 1.956608425 | 5.199754744 | 1.698102342 |
| SsWRKY42 | 3.451096137 | 1.430479653 | 0.079412524 | 1.024058928 | 1.567826042 |
| SsWRKY43 | 10.3849762 | 3.147316049 | 6.163368724 | 9.631554585 | 5.438516961 |
| SsWRKY44 | 1.920446864 | 1.549654578 | 8.027430485 | 6.884591477 | 0.757089181 |
| SsWRKY45 | 13.86267006 | 8.796465026 | 36.14972095 | 28.03295219 | 10.68714717 |
| SsWRKY46 | 7.386578267 | 2.183732499 | 9.972843795 | 4.460870398 | 0.283216225 |
| SsWRKY47 | 6.584620138 | 0.266483658 | 1.155686737 | 1.673240834 | 0.936747757 |
| SsWRKY48 | 30.35885607 | 0.28580145 | 2.592629176 | 2.364536224 | 0.33637968 |
| SsWRKY49 | 4.663338253 | 6.275321983 | 7.817583908 | 10.01338277 | 4.660490669 |
| SsWRKY50 | 2.593436353 | 0.92296228 | 2.303896806 | 0.928562136 | 0 |
| SsWRKY51 | 6.996183497 | 10.99441237 | 8.525537666 | 7.966199547 | 6.481220269 |
| SsWRKY52 | 0.857058931 | 3.713323235 | 92.71596994 | 12.399115 | 0.045884193 |
| SsWRKY53 | 17.36426824 | 3.797026368 | 23.91047321 | 3.022592498 | 1.012279839 |
| SsWRKY54 | 0.553545649 | 0.157462063 | 1.896893656 | 1.870937713 | 0 |
| SsWRKY55 | 58.31806722 | 18.51337544 | 63.73719829 | 77.21264486 | 14.73757661 |
| SsWRKY56 | 40.96349931 | 32.54145738 | 44.32432981 | 24.54110636 | 7.489235647 |
| SsWRKY57 | 2.731119036 | 29.89881865 | 1.685505222 | 3.217956573 | 0.544526222 |
| SsWRKY58 | 22.34379905 | 1.783894154 | 2.698422642 | 4.006773385 | 1.110526717 |
| SsWRKY59 | 27.91201066 | 0.107912145 | 18.24168435 | 1.074514636 | 0 |
| SsWRKY60 | 2.91028412 | 2.779980632 | 9.954255827 | 5.233826672 | 3.605826079 |
| SsWRKY61 | 16.87597599 | 0.055916015 | 6.531764916 | 2.423600446 | 0.666889486 |
| SsWRKY62 | 7.427545544 | 4.265743424 | 3.882791121 | 4.86256015 | 0.463444731 |
| SsWRKY63 | 30.19629752 | 1.645455837 | 4.807265197 | 6.200725171 | 3.671083032 |
| SsWRKY64 | 3.414120107 | 0.068118079 | 1.217658697 | 4.016438913 | 1.941966347 |
| SsWRKY65 | 30.57897227 | 29.13303291 | 46.85012131 | 27.30990281 | 26.30972961 |
| SsWRKY66 | 6.232740951 | 0 | 0.019714148 | 0.277333627 | 0 |
| SsWRKY67 | 5.205839432 | 8.566958329 | 188.1588802 | 82.06707055 | 26.10810571 |
| SsWRKY68 | 5.504974457 | 1.614143818 | 13.7807117 | 14.45859176 | 7.906232363 |
| SsWRKY69 | 2.952091873 | 0.584775313 | 6.34013618 | 7.912142444 | 3.76250381 |
| SsWRKY70 | 0.387791144 | 0.031060766 | 0.488846529 | 0.636756361 | 0 |
| SsWRKY71 | 18.87603649 | 0.032108829 | 0.673788468 | 1.071994122 | 0 |
| SsWRKY72 | 3.43600759 | 1.281416935 | 0.107991662 | 0.144685643 | 0.048455873 |
| SsWRKY73 | 11.41967228 | 0.020524467 | 0.095710257 | 0.096173398 | 0.016104452 |
| SsWRKY74 | 0.062098178 | 0 | 0 | 0.067006056 | 0 |
| SsWRKY75 | 32.72613857 | 0 | 0.011195466 | 0.348738857 | 0.030140442 |
| SsWRKY76 | 12.7178554 | 11.37900011 | 24.96663885 | 13.41204754 | 7.183664353 |
| SsWRKY77 | 6.950775666 | 2.680385774 | 1.244001039 | 0.40179238 | 0.478442943 |
| SsWRKY78 | 4.776573148 | 4.534374921 | 4.065134063 | 6.407990046 | 1.159338686 |
| SsWRKY79 | 0.318615885 | 0 | 0.228094538 | 0.534795996 | 0 |
| SsWRKY80 | 20.06416781 | 14.00940358 | 20.35765315 | 14.16285087 | 5.915417893 |
| SsWRKY81 | 97.74521407 | 9.806740275 | 30.58872796 | 8.941969271 | 8.076837448 |
| SsWRKY82 | 30.51559257 | 3.478897132 | 5.258082261 | 9.431003809 | 1.038737153 |
| SsWRKY83 | 0.063309006 | 0.029157321 | 0.169959022 | 0.03415629 | 0.045756382 |
| SsWRKY84 | 36.4958157 | 30.01960691 | 43.43513135 | 46.07517915 | 15.87715838 |
| SsWRKY85 | 70.06371207 | 8.83315066 | 28.60233438 | 17.13960106 | 4.915749266 |
