# Supplementary material for: Genome-wide identification and characterization of WRKY gene family in Salix suchowensis
Source: PeerJ. 2016 Sep 7;4:e2437. doi: 10.7717/peerj.2437 (PMC5018666; doi:10.7717/peerj.2437)
Supplement: Supplemental Information 8 [file peerj-04-2437-s008.docx]

**Table 3. The number of WRKY genes identified in *Arabidopsis thaliana*, *Cucumis sativus*, *Poplulus trichocarpa*, *Vitis vinifera*, *Salix suchowensis* and *Oryza sativa*.**

| Species | Group | | | | | | |
| --- | --- | --- | --- | --- | --- | --- | --- |
|  | I | IIa | IIb | IIc | IId | IIe | III |
| *Arabidopsis thaliana* | 13 | 4 | 7 | 18 | 7 | 9 | 14 |
| *Cucumis sativus* | 10 | 4 | 4 | 16 | 8 | 7 | 6 |
| *Populus trichocarpa* | 50 | 5 | 9 | 13 | 13 | 4 | 10 |
| *Vitis vinifera* | 12 | 4 | 8 | 16 | 7 | 6 | 6 |
| *Salix suchowensis* | 19 | 4 | 8 | 23 | 13 | 11 | 7 |
| *Oryza sativa* | 34 | 4 | 8 | 7 | 11 | 0 | 36 |
